# Tumor Microbial Communities and Thyroid Cancer Development—The Protective Role of Antioxidant Nutrients: Application Strategies and Future Directions

**DOI:** 10.3390/antiox12101898

**Published:** 2023-10-23

**Authors:** Francesca Gorini, Alessandro Tonacci

**Affiliations:** Institute of Clinical Physiology, National Research Council, 56124 Pisa, Italy; alessandro.tonacci@cnr.it

**Keywords:** antioxidant nutrients, gut–thyroid axis, microbiota, oxidative stress, psychophysiology, sensory analysis, sensory features, technologies, thyroid cancer, tumor microbiome

## Abstract

Thyroid cancer (TC), the most frequent malignancy of the endocrine system, has recorded an increasing incidence in the last decades. The etiology of TC remains at least partly unknown and, among modifiable risk factors, the gut microbiota and dietary nutrients (vitamins, essential microelements, polyphenols, probiotics) have been recognized to not only influence thyroid function, but exert critical effects on TC development and progression. Recent discoveries on the existence of tumor microbiota also in the TC microenvironment provide further evidence for the essential role of tumor microorganisms in TC etiology and severity, as well as acting as prognostic markers and as a potential target of adjuvant care in the treatment of TC patients. Therefore, in this review, we summarize current knowledge on the relationship of the tumor microbiome with the clinical tumor characteristics and TC progression, also illustrating the molecular mechanisms underlying this association, and how antioxidant nutrients may be used as a novel strategy to both control gut health and reduce the risk for TC. Furthermore, we discuss how new technologies might be exploited for the development of new foods with high nutritional values, antioxidant capability, and even attractiveness to the individual in terms of sensory and emotional features.

## 1. Introduction

Thyroid cancer (TC), the most frequent malignancy of the endocrine system, currently ranks in the United States as the 13th most common cancer diagnosis overall and the sixth most common among women [1,2]. Among the histological types, papillary thyroid carcinoma (PTC) accounts for approximately 90% of all cases, followed by follicular thyroid carcinoma (FTC) (4%), Hürthle-cell carcinoma (2%), medullary thyroid carcinoma (2%), and anaplastic thyroid carcinoma (ATC) (1%) [2]. In 2020, the estimated number of new cases of TC was approximately 449,000 in women and 137,000 in men globally, with most countries having an age-standardized incidence rate that is about three times higher in women (10.1 per 100,000) than in men (3.1 per 100,000) [3]. Since the mid-1970s to 2013, the incidence of TC has markedly risen in the United States, with an annual rate increase of 3%, mainly owing to small (<2 cm) PTCs [4]. A similar trend was also observed in other developed countries while, in contrast, mortality rates remained stable or declined in most territories [3,5]. These epidemiological features have been largely attributed to overdiagnosis [3]. Indeed, the changes in clinical practice guidelines recommended by the American Thyroid Association, including the reclassification of the non-invasive encapsulated follicular variant subtype of PTC (FVPTC) from a malignant to an in situ neoplasm (“non-invasive follicular thyroid neoplasm with papillary-like nuclear features”), having an extremely low risk of adverse outcomes, like tumor recurrence or spread, coincided with a decline of FVPTC incidence by 10% in recent years [6,7]. On the other hand, a continuous increase in the incidence of larger classical PTC and other PTC variants was recorded over time, indicating that the reasons underlying TC incidence trends were multifactorial [6]. Although more than 95% of TC cases belong to differentiated TC (PTC, FTC, and Hürthle-cell carcinoma), deriving from thyroid follicular epithelial cells and characterized by an excellent prognosis [8,9], the etiology of TC is not fully clarified [10]. While childhood exposure to ionizing radiation, a history of benign thyroid nodules and goiter, and a family history of proliferative thyroid disease are established risk factors for TC, the role of other modifiable factors, such as dietary patterns and microbiota composition in TC carcinogenesis, have been recently explored [2,11,12,13]. Deficiency of iodine, considered a trace element essential for the formation of thyroid hormones, has been associated with an increased risk of TC, promoting the development of FTC and ATC, while the effect of iodine supplementation, though still controversial, may influence the ratio of PTC to FTC, suggesting that an excessive iodine intake could act as a risk factor for PTC [11,14]. However, other nutritional factors, like selenium (Se), zinc (Zn), and flavonoids, not only play a crucial role in the thyroid gland, but, thanks to their antioxidant properties, might exert protective effects against impaired redox homeostasis, the signature of certain thyroid pathologies, including TC [15]. Growing evidence supports the contribution of increased production of reactive oxygen species (ROS) in the pathogenesis and progression of TC [16,17], with oxidative stress inversely correlated with tumor differentiation and directly correlated with the presence of somatic mutations and with worse TC presentation and higher TC aggressiveness [17].

Furthermore, a great deal of attention has recently arisen towards the microbiota, defined as the living microorganisms found in a defined environment and located in various districts of the human body (gut, skin, lung, oral cavity) [18]. Microbiota are complex systems consisting of trillions of microorganisms, predominantly bacteria, whose alterations in composition have been linked to disease development and progression, including heart disease, liver disease, chronic kidney disease, brain disorders, diabetes, inflammatory bowel disease, respiratory disease, and cancers [18,19]. A number of studies have revealed a close correlation between the microbiota of the gut, the largest endocrine organ, and thyroid disease; in fact, if the thyroid hormone may influence the gastrointestinal structure and function, gut microbial alteration, namely, gut dysbacteriosis, bacterial overgrowth, and increased gut permeability, favor the development of autoimmune thyroiditis (Hashimoto’s and Graves’ diseases) and TC pathogenesis (e.g., [20,21,22,23,24]). Importantly, dietary components exert significant impact on the microbiota composition and function [19]. Thus, while the processed food highly used in Western diets contains numerous additives that might lead to microbial dysbiosis due to oxidative stress, recognized as the main mechanism of toxicity in humans [25], food antioxidants, such as polyphenols, vitamins, Zn, and Se, capable of stopping the production of ROS, along with stabilizing and scavenging the prevailing ROS in the host body, show excellent beneficial influence on the gut microbiota [19,26].

Of note, in recent years, commensal bacteria and other microorganisms—the tumor microbiome, defined as the ecosystems created by resident microbes, their genomes, and functional interactions within the tumor microenvironment—have also been found in a variety of cancer tissues, including breast, lung, colorectal, and prostate cancers [27]. As regards TC, so far, only a few studies have characterized the microbial diversity and composition of tumor tissues.

Therefore, in this review, we summarize current knowledge about the existing evidence on the association between microbiota and TC, focusing on the emerging role of the microbial communities in TC, discussing its relationship with the clinical tumor characteristics and cancer progression, and exploring the potential of the microbiome for early diagnosis and improved treatment of TC. Furthermore, given the accumulating evidence on the existence of a thyroid–gut axis and the relevant correlations between the composition of intestinal bacteria and TC, we illustrate the molecular mechanisms underlying this interaction and the link with TC carcinogenesis, as well as how antioxidant nutritional and dietary elements may be used as novel strategies to control gut health and, potentially, to prevent TC. We complement the investigation with some new hints potentially useful to develop and optimize strategies based on food, to make the dietary supplementation not only favorable in terms of the effects on the oxidative stress mechanisms, but also attractive to the individual in terms of sensory and emotional features.

## 2. The Microbiota and Thyroid Axis

The microbiota, which collectively refers to microorganisms (bacteria, viruses, fungi, etc.) resident in the human body, plays a vital role in both the maintenance of normal physiology and the occurrence of clinical outcomes [27,28]. The gut microbiota consists of almost 1200 bacterial species (at least 160 such species in each individual) and around 90% of the total human cells, whose gene count exceeds the human genome’s gene count by ~100-fold [13,29,30]. The community of gut bacteria, most of which are strictly anaerobic and represent a mass of approximately 1.5–2 kg, is mainly composed (>90%) of Bacteroidetes, Firmicutes, Actinobacteria, Proteobacteria, and Verrucomicrobia, with Firmicutes and Bacteroidetes accounting for almost 90% of the population of the total gut microbiota [19,31,32].

**Figure 1:**
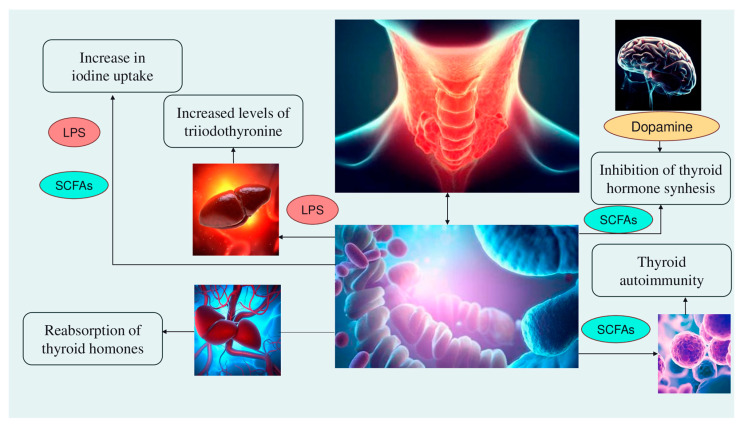
The mechanisms involved in the microbiota–thyroid axis. Abbreviations: LPS: lipopolysaccharide; SCFAs: short chain fatty acids.

If, on the one hand, health depends on nutritional, metabolic, and immune functions of the microbial communities that are in symbiosis with the host, on the other hand, gut microbiota dysbiosis, a condition characterized by an alteration in the composition and physiological functions of the gastrointestinal microbiota as a consequence of diseases, changes of dietary habits, stress, or antibiotic use, may increase the prevalence of type 2 diabetes, cardiovascular disease, autoimmune disease, inflammatory bowel disease, and central nervous system disorders [18,29,33,34]. A gut-endocrine–homeostasis-thyroid axis has been shown in recent studies, which reported an altered composition of the gut microbiota in patients with Hashimoto’s disease and Graves’ disease, further suggesting that microbiota analysis could provide an alternative non-invasive diagnostic methodology for thyroid diseases [21,23,24,34,35]. The intestine, in fact, is a target organ of thyroid hormones, namely, triiodothyronine (T3), whose actions mostly depend on its interaction with nuclear thyroid receptor (TR) alpha 1, the main TR isoform expressed in the intestine epithelial cells [36,37]. On the other hand, the gut microbiota plays a key role in both the homeostasis of thyroid function and thyroid disease pathogenesis via different mechanisms (see [32] for more details):

1. Alteration of iodine uptake, the main rate-limiting step in thyroid hormonogenesis, affecting the activity of sodium iodide symporter (NIS) through two processes: (a) The binding of the Gram-negative bacterial endotoxin lipopolysaccharide (LPS), released by the gut microbiota, to the thyroid cell toll-like receptor 4 (TLR-4). TLR-4 in turn activates the nuclear factor kappa-light-chain-enhancer of activated B cells (NF-kB), which subsequently promotes NIS transcription through paired box 8 (PAX8) [38]. (b) Alternatively, enhancement of NIS expression may also occur through histone deacetylase (HDAC) inhibition by an important metabolite of the gut microbiota, butyrate (which belongs to the class of short-chain fatty acids—SCFAs; see later in Section 6) [39,40];
2. Modulation of activities of iodothyronine deiodinases, enzymes responsible for the conversion of thyroxine (T4) to its active form T3 by type 1 and type 2 deiodinases (D1, expressed mostly in the liver, kidney, thyroid, and pituitary, and D2, expressed primarily in the thyroid, central nervous system, pituitary, developing cochlea, brown adipose tissue, and skeletal muscle [41]) or to reverse T3, its inactive form, by type 3 deiodinase—D3 [42]. This occurs through a complex thyroid–gut axis pathway involving LPS capable of inducing the decrease in D1 activity in the liver [43] and, at the same time, activating D2 in the mediobasal hypothalamus, ultimately promoting the conversion of T4 to T3 [44];
3. Modulation of T3 and T4 bioavailability through the deconjugation of sulfoconjugated and glucuroconjugated iodothyronines by bacterial sulfate esterase or β-glucuronidase, respectively, thus inducing the reabsorption of thyroid hormones in the enterohepatic circulation. In humans, a recycling mechanism has been described for steroids hormones, biliary acids, and vitamins, while as for thyroid hormones, direct proof has been only established in animal models [31];
4. Regulation of the SCFAs-mediated balance between T helper 17 (Th17) cells and regulatory T cells (Treg), two subtypes of CD4+ lymphocytes exerting opposite effects (release of pro-inflammatory cytokines, i.e., interleukin—IL-17 or anti-inflammatory IL-10, respectively), in autoimmune inflammatory diseases and immune tolerance [35,45]. All these immune cells play a role in the pathogenesis of autoimmune thyroid disease (AITD), like Hashimoto disease (HD) and Graves’ disease. For instance, *Prevotella* is correlated with reduced proinflammatory Th17 polarization and increased differentiation of anti-inflammatory Treg; therefore, it has been speculated that the Th17/Treg homeostasis regulation might be a potential pathogenic pathway for *Prevotella* in HT patients [32,46];
5. Involvement of the microbiota–gut–brain signaling in dopamine release, synthesis, and bioavailability. Certain species up-/downregulate the system dopamine transporter/dopamine binding efficiency, while others are positively or negatively correlated with the activity of tyrosine hydroxylase, an enzyme involved in dopamine synthesis [47,48]. Furthermore, butyrate’s intrinsic HDAC inhibitor activity influences neurotransmitter levels [47]. Since dopamine inhibits synthesis and secretion of the thyroid-stimulating hormone (TSH), thyroid function may be affected [35] (Figure 1).

Therefore, although further data are still required to elucidate the specific relationships and mechanisms between the gut microbiota and the thyroid, intestinal microorganisms appear to act directly or indirectly on the gland by mainly influencing iodothyronine synthesis, conversion, and storage, as well as through immune regulation [32].

## 3. The Association between Microbiota and Thyroid Cancer

The gut microbiota has been associated with the development, diagnosis, and treatment of various tumors (e.g., hepatocellular carcinoma, pancreatic, gastric, and breast cancers) [27,49,50], with evidence supporting a causal role for gut dysbiosis in the development of colorectal cancer [51,52,53]. Despite the multifactorial etiology of cancer, which is the result of a complex interaction between genetic alterations and environmental factors, it has been estimated that approximately 15% of malignancies worldwide are caused by infections with oncogenic pathogens [34,54]. Besides the “oncomicrobes”, 11 microorganisms (7 viruses, 1 bacterium, and 3 parasites, e.g., Epstein–Barr virus, Hepatitis B/C virus, Human Papillomaviruses, Human Immunodeficiency Virus, and *Helicobacter pylori*—Hp, labeled as Group 1 carcinogens [55]) of the estimated ~10^30^ distinct microbial species living on Earth [56], an increasing amount of evidence supports the existence of another category of microorganisms, which is not causally related to cancer but able to promote tumor development and modulate both tumor progression and responses to numerous forms of cancer therapy [30,57]. Although studies on the role of the microbiota in cancer are in their infancy, the technological advancement with the application of 16S ribosomal ribonucleic acid (rRNA) gene sequencing, has allowed establishing a close relationship between the activity of intestinal microorganisms, particularly their metabolites, and the protective or promoting effects against cancers [34,49].

So far, only a few studies have investigated the relationship between microbiota and TC (Table 1). Overall, the research performed demonstrated a significant difference in the richness, diversity, and composition of intestinal microbial communities between TC patients and healthy controls, suggesting the predictive value of the gut microbiota in discriminating TC statuses [20,34]. Feng et al. [20], analyzing 30 TC patients and 35 healthy controls’ fecal samples by 16S rRNA gene sequencing, found that the TC group had a greater gut microbiota richness and diversity (α-diversity) compared to the control group, with TC patient samples enriched in the abundance of Firmicutes and Proteobacteria, and samples of controls enriched in Bacteroidetes. Furthermore, β-diversity, defined as the extent of similarity between two microbiota communities, was also significantly different between TC and control individuals, for a total of 21 different genera, 5 of them more effective in distinguishing TC patients from controls [20]. Of note, many genera of Proteobacteria, such as *Enterobacter* and *Haemophilus*, were also increased in fecal samples from patients with primary liver cancer [58]. Similar results were found by Zhang et al. [59], who compared the gut microbiome of 20 patients with TC, 18 individuals with thyroid nodules, and 36 matched healthy controls, showing higher microbial abundance and distinct composition in individuals with thyroid disease than in the healthy control group. In particular, the gut microbiome of the TC group was characterized by the relative dominance of *Neisseria* and *Streptococcus*, which have also been associated with the development of gastric cancer in the absence of Hp infection [60], *Streptococcus* accounting for the largest proportion in Hp-negative gastric cancer at the family level [61] and related to colorectal carcinogenesis, as well [62], supporting the potential of these bacteria possibly playing a role in thyroid disease. Additionally, both studies found a lower relative abundance of certain genera, including *Butyricimonas*, *Lactobacillus*, *Bacteroides*, and the Lachnospiraceae family [20,59]. Overall, these microorganisms are known to participate in the production of SCFAs (acetate, propionate, butyrate, and valerate), the main metabolites produced in the colon by bacterial fermentation of dietary fibers and resistant starch, which in turn play an essential role in the modulation of gut microbiota physiology and composition by regulating immunity and suppressing or promoting inflammatory responses, as described thereinafter in the text [63,64,65,66]. Importantly, some *Lactobacillus* strains can fix inorganic selenite into selenoproteins [67], such as glutathione peroxidase (GPx) and thioredoxin reductase (TrxR), key factors for oxidative stress control [68], and iodothyronine deiodinases, essential enzymes for thyroid function [69]. Furthermore, altered gut microbiota genera in the TC group were significantly associated with both serum lipid (e.g., lipoprotein A, apolipoprotein A and B) and lipid metabolite levels (e.g., linolenic acid, gamma-aminobutyric acid) [20], confirming that dysregulation of lipid metabolism represents an important metabolic alteration in cancer, including TC [70,71,72] and disease indices, namely, increased serum TSH levels [59], recognized as positively associated with the incidence of nodular goiter and PTC [73]. Previously, the study by Shen et al. [71] revealed that the serum of patients with distant metastatic PTC (n = 37) was characterized by an elevated concentration of gamma-aminobutyric acid (GABA), which could be implicated, along with its receptors, in the oncogenesis/metastasis of various tumors [74,75,76]. These subjects also had increased levels of serum aminooxyacetic acid, a nonselective inhibitor of transaminases, including GABA transaminase [77], thereby increasing GABA concentration, and of 4-deoxypyridoxine, a potent antagonist of vitamin B6 coenzyme (that is involved in the regulation of immune responses) [78]. Since both are not endogenous metabolites, they could be related to diet–gut microbiota interactions, suggesting that serum metabolomics profiling could significantly discriminate PTC patients according to distant metastasis [71]. In contrast to prior findings, a recent cross-sectional study [79] reported a reduced richness and diversity of the gut microbiota in stool samples of TC patients (n = 60) compared to those of healthy controls (n = 60), probably because of differences in the demographics of controls, dietary habits, and tumor TNM status. In addition, although there was no significant difference in the Firmicutes/Bacteroidetes ratio (accepted to have an important influence on maintaining normal intestinal homeostasis [80]), between the two groups, about 70% of TC patients showed a relatively higher abundance of Proteobacteria, a signature of microbial dysbiosis possibly related to obesity [81], in accordance with Feng and co-workers [20]. Notably, a four-genus microbial signature was able to distinguish TC patients with metastatic lymphadenopathy from those without it; however, no significant difference in gut microbiota richness or diversity was observed between the two groups [79]. Furthermore, the authors detected deficient genetic information processing related to five categories in the TC group [79] (Table 1). Overall, these results, albeit needing to be supported by animal models, provide relevant information on the potential role of the gut microbiome in TC pathogenesis and how it might be important to prevent and regulate intestinal dysbiosis.

### Microbial Communities in Thyroid Cancer Tissues

Although the presence of bacteria in tumor tissues, traditionally considered sterile, dates back to more than 100 years ago, only thanks to a combination of imaging, sequencing, and cultivation techniques, and genetically engineered and germ-free animal models (grown in sterile conditions and completely free of intestinal bacteria [82]), was it possible to exclude the possibility of contamination and detect the very low microbial content in tumors [30,83]. Thus, in recent years, tumor-type specific bacteria have been observed in a variety of tumors, e.g., melanoma, colorectal, pancreatic, gastric, breast, lung, ovarian, prostate, and bladder cancers, suggesting their involvement in processes related to tumorigenesis and cancer progression [27,28]. Nejman et al. [84], analyzing the tumor microbiome of 1526 tumors and their adjacent normal tissues across seven cancer types, found that the intratumor bacteria are mostly intracellular and are present in both cancer and immune cells, with the phyla Proteobacteria and Firmicutes representing the majority of bacterial sequences detected in all tumor types, while the Actinobacteria phylum dominates in non-gastrointestinal tumors. The tumor microenvironment, recognized as a pivotal player in tumorigenesis, consists of both proliferating malignant cells and non-malignant components, including tumor stromal cells (stromal fibroblasts and immune cells, such as microglia, macrophages, and lymphocytes), elements of the extracellular matrix, and endothelial cells [28,85]. If genetic/epigenetic alterations promote the process of tumor initiation and progression [28,85], the microbiome and its metabolites, despite their low biomass, can influence the components of the tumor microenvironment by modulating the processes of inflammation, proliferation, and cell death, therefore playing a key role in shaping tumor development [86].

Compared to other tumors, the intratumoral microbiome of TC has been poorly explored; however, it can be hypothesized that the thyroid can be colonized by microorganisms, since gastric mucosal cells and thyroid follicular cells derive from primitive gut cells during embryonic development (Table 2). The study by Liu et al. [1], including 93 sample tissues (divided into tumor, paratumor, and normal tissues) and stool samples from 25 TC patients (19 malignant cases, 6 benign cases), reported a higher α-diversity of fecal samples than that of thyroid tissues, while the total number of microorganisms in tissue samples decreased with the increasing distance from the cancerous tissue. The predominance of Proteobacteria, and, in particular, of *Pseudomonas mucidolens*, was found in all three types of tissue samples, especially in patients with malignant TC, suggesting that they might participate in TC development, but not in stool samples that were instead characterized by the predominance of Firmicutes [1]. While other *Pseudomonas* species have been associated with bacteremia, nosocomial infections, and cystic fibrosis [87,88], *Pseudomonas aeruginosa* has recently been shown to enter cancer cells and induce apoptosis, without any effect on normal cells [89]. The phylum Proteobacteria consists of facultative anaerobic bacteria, which are not dominant in the healthy intestine, where over 90% of the gut microbiota is characterized by strict anaerobes [90]. Moreover, since the authors observed only a very partial overlap of sequences and metabolic pathways (especially in fatty acid degradation) between the thyroid and intestine, excluding a linkage between gut and thyroid microbes, the abundance of Proteobacteria in thyroid tissues could be caused by TC cells, which possibly give rise to a unique microbial community [1]. Another research study [12], characterizing tumor tissues and matched peritumor (approximately 3 cm adjacent to the cancer tissue) tissues from 55 TC patients at the early stage (stages I and II) who underwent thyroidectomy, reported significantly lower α-diversity and richness in tumors than in peritumor tissues, consistent with Yu et al. [79] and with that observed for other cancers [91,92]. Furthermore, microbial diversity and composition were significantly different between tumor and peritumor microenvironments: while *Sphingomonas*, which has also been identified as the dominant genus in thymic epithelial tumors [93], colitis-associated cancer [94], and gastric mucosa-associated lymphoid lymphoma [95], predominated in tumor tissue, *Comamonas*, also associated with lymph node metastasis in pancreatic cancer [96], had higher abundance in peritumor tissues. The combination of these genera could therefore serve both as a signature to distinguish tumors from peritumor tissues and as a prognostic marker in cancer progression in patients with early-stage TC [12]. Of note, the authors also reported higher α-diversity of the thyroid microbiome from patients at the N1 stage in comparison to those at N0, but no significant differences in α-diversity and richness between female and male patients [12]. Gnanasekar and co-authors [97], based on data of microbial sequences obtained from the Genomic Data Commons legacy archive for a total of 563 TC patients, as well as confirming that the tumor tissue contained lower microbe abundance than the adjacent normal tissue, as in Dai et al. [12], found heterogeneity in the composition of the carcinoma microbiome between males and females, and between PTC (classical, follicular variant, and tall cell—TCPTC) subtypes. The aggressiveness of TCPTC could depend on the dominance of *Micrococcus lutheus*, which has been associated with infections in severely immunocompromised patients [98,99], and of *Bradyrhizobium* sp. *BTAi1*, correlated with a lower free-survival probability in cervical cancer cases [100]. At the same time, the microbe abundance in male samples was related to a greater number of chromosomal alterations and inversely associated with tumor suppressive pathways, explaining the worse prognosis of TCPTC in males than in female patients [101]. Of interest, *Frankia* sp. and *Anabaena* sp. *K119*, both enriched in normal tissue samples of all PTC subtypes, were inversely correlated with the pathologic M stage [97]. A recent study [82] investigated the role of the PTC tumor microbiome in cancer progression, showing that the tumor bacterial α-diversity was significantly higher in patients with advanced lesions (T3 or T4) than those with relatively mild lesions (T1 or T2). In contrast to Dai et al. [12], the α-diversity was higher in females (who are expected to have a greater PTC incidence compared to male patients), indicating that the microbiome presents specific characteristics that vary by sex, in addition to tumor staging. The authors further observed significant differences in β-diversity, with *Pseudomonas*, the most abundant genus in all groups, presenting higher relative abundance in patients with T1 and T2 PTC than in those with T3 or T4 PTC. Moreover, an interaction between intratumoral bacteria and AITD-related antibodies was found. In particular, Prevotellaceae, *Bacteroides*, and *Bifidobacteria* showed a negative relationship with anti-thyroperoxidase (TPO) levels, corroborating previous findings [102] and suggesting a role of these microbial genera in the pathogenesis of AITD by molecular mimicry [82]. These immunoregulatory effects of the tumor microbiome may in turn enhance or impair the immune response against the tumor and, consequently, affect the final outcome of PTC [82]. Hence, the TC microbiome appears to play a crucial role not only in the tumor progression, but, by interacting with autoimmune antibodies, might also contribute to tumor invasion.

## 4. Oxidative Stress in Thyroid Cancer

If, at physiological concentration, ROS are considered as essential second messengers in cells, participating in proliferation, gene expression, host defense, and hormone synthesis, when in excess, they are responsible for alterations in the chemical structure and, consequently, in the function of proteins, lipids, and DNA, leading to genotoxic responses up to cell death, through the apoptotic pathway [103,104,105,106]. For instance, the 2′-oxidized form of guanine, 8-oxo-deoxyguanosine, is a valuable marker of oxidative DNA damage during carcinogenesis, and its prognostic significance has been demonstrated in solid tumors, including TC [107,108]. Oxidative stress, defined as an imbalance caused by excessive production of pro-oxidant substances, such as ROS (e.g., superoxide anion, hydroxyl and peroxyl radicals, hydrogen peroxide—H_2_O_2_) and reactive nitrogen species (RNS, e.g., peroxynitrite, nitrosoperoxycarbonate), and/or by inefficient functioning of antioxidant systems [15], has been recently linked to TC development and progression [105,109,110]. ROS are mainly produced at sites on the mitochondrial complexes I and III of the electron transport chain, as a byproduct of respiration, and also through a number of enzymatic systems, including nicotinamide adenine dinucleotide phosphate oxidases (NOXs), xanthine oxidases, nitric oxide (NO) synthase, and cytochrome P450 reductase [103,105]. Thyroid cells are thought to have a relatively high tolerance to oxidative stress compared to many other cell types [110], due to the involvement of ROS in the initial stages of thyroid hormone synthesis during dietary iodide oxidation by TPO- and H_2_O_2_-generating enzymes, the dual oxidases (DUOXs) 1 and 2 [15,111]. Both DUOX1 and -2, members of the NOX family, are expressed in the thyroid, though the expression level of DUOX2 is fivefold higher compared to DUOX1, and the role of the latter in the gland remains largely unknown [112,113]. As for DUOX2, unlike other ROS-producing enzymes, its function and expression are tightly regulated [112]: in fact, it is restricted to the apical membrane–luminal interface in an iodination complex, the thyroxisome, where H_2_O_2_ is consumed by TPO, which decreases the availability of H_2_O_2_ at the apical membrane of thyrocytes and the possible oxidative damage of this substance [114]. Additionally, the thyroid redox homeostasis is guaranteed by antioxidant enzymes, including GPx, TrxR, and catalase (CAT), with GPx and TrxR more effective in H_2_O_2_ elimination at pathophysiological levels [115]. A recent study reported higher GPx activity in most tumors than in normal tissues, with a significant inverse correlation with H_2_O_2_ generation in all benign and malignant lesions, possibly suggesting an attempt of antioxidant defenses to counteract the increased ROS production [17]. Conversely, previous research showed a decrease in the expression of GPx1 and TrxR1 in TC cells compared to healthy cells, highlighting the inability of tumor tissues to produce an effective antioxidant system against the enhanced generation of free radicals [104]. Thyrocytes also express NOX4, a constitutive enzyme producing H_2_O_2_ and hydroxyl radical in intracellular compartments and mainly regulated at the transcriptional level [116]; however, NOX4 function in physiological thyroid hormonogenesis needs to be better elucidated [113].

Although the role of DUOX enzymes in tumorigenesis is ambiguous, in the human thyroid cell line and primary thyrocytes, DUOX1 expression, induced via the IL-13 pathway in response to ionizing radiation, is the primary source of long-term ROS production that causes persistent DNA damage and potential genomic instability [117]. Previously, H_2_O_2_ was reported to cause *RET/PTC1* rearrangement (a signature of radiation-induced PTC) in thyroid cells, suggesting that oxidative stress contributes to *RET/PTC1* formation found in thyroid lesions, even in the absence of radiation exposure [118]. DUOX1 appeared to be overexpressed even in sporadic tumors, indicating a role of H_2_O_2_ in the initiation of PTC [117,119], consistent with prior studies observing no difference in DUOX1 expression in both radiation-induced PTC from the Chernobyl Tissue Bank and in sporadic PTC [119,120]. As shown by Muzza and co-workers [121], the whole intracellular DUOX and NOX4 activity is significantly higher in PTC cells than in normal thyroid cells, corroborating the upregulation of NOX4–p22^phox^ (that is required for NOX4 catalytic activity to form a heterodimeric enzyme complex) in thyroid tumors, which might be related to a higher proliferation rate and tumor progression [122]. In PTC, NOX4 expression is significantly higher in the presence of the *BRAF^V600E^* mutation [123], the most frequent genomic alteration found in PTC, present in around 40–60% of cases and associated with increased cancer-related mortality and thyroid cell dedifferentiation, as well [124,125]. NOX4 upregulation is controlled at the transcriptional level by the BRAF^V600E^ oncogenic protein via the transforming growth factor (TGF)-β/Smad3 signaling pathway, leading to constitutive activation of mitogen-activated protein kinase (MAPK) and downregulation of thyroid-specific genes [113,123]. Interestingly, BRAF^V600E^-positive tumors are often associated with a significant decrease or a complete loss of NIS expression, responsible for the resistance of this type of tumor to radioiodine treatment, which may occur via two potential mechanisms: (1) BRAF-induced TGF-β1 represses *NIS* gene expression through Smad3, a NOX4-dependent downstream pathway; (2) ROS, deriving from NOX4 upregulation, induces Smad3 to inhibit PAX8, the major regulator of *NIS* transcription, through redox-sensitive epigenetic modifications [123,125], namely, hypermethylation of the *NIS* promoter [126]. NOX4 is further involved in another relevant process in thyroid carcinogenesis related to metabolic adaptation of PTC cells, characterized by a hypoxic microenvironment [113,127]. Indeed, NOX4 stabilizes the nuclear hypoxia-inducible factor-1 alpha (HIF-1α), which triggers a complex transcriptional response implying the shift of cellular glucose metabolism towards glycolysis (the Warburg effect) and the concomitant suppression of oxidative metabolism, in order to support the biosynthetic requirements of uncontrolled proliferation [127,128]. Importantly, in poorly differentiated TC (PDTC), the presence of a high density of tumor-associated macrophages (TAMS) has been found to correlate with invasion and decreased cancer-related survival [129]. NOX4 is upregulated in a variety of type cancers, and in lung cancer, tumoral NOX4 recruits TAMs via ROS/phosphatidylinositol 3-kinase signaling-dependent various cytokine production, a process that contributes to cancer malignant progression [125,130]. Therefore, we might speculate an interaction between NOX4 and the tumor microenvironment even in TC through the recruitment of tumor-associated inflammatory cells, including TAMs. Muzza et al. [17] recently reported that ROS generation and NOX4 expression were higher in malignant and benign lesions than in normal tissues, and, among malignant lesions, a significant association was detected between oxidative stress and the aggressiveness of the tumor (i.e., increased levels of H_2_O_2_ in FTC and PDTC/ATC compared with PTC). Furthermore, a significantly increased H_2_O_2_ concentration was found in mutated PTCs (including *BRAF* and *RAS* mutations and *RET* fusion, genomic alterations mutually exclusive and resulting in the constitutive activation of the MAPK signaling pathway [131,132]) compared to in non-mutated PTCs, indicating that oxidative stress might have both prognostic and therapeutic relevance in TC [17].

## 5. The Dual Relationship between the Microbiota and Cancer: The Biological Mechanisms

**Figure 2:**
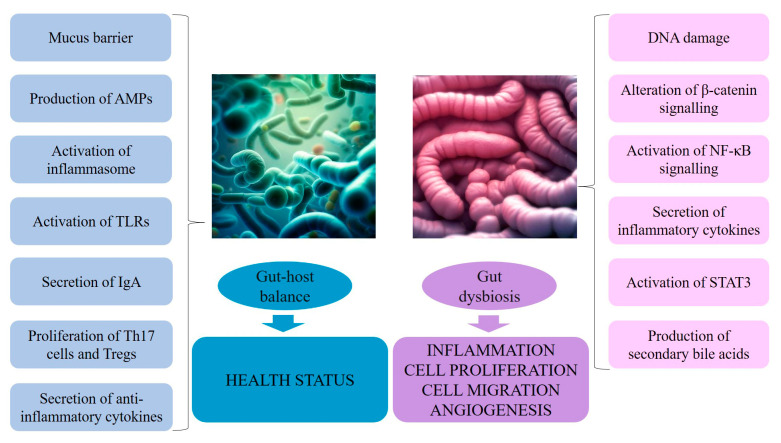
The gut microbiota status and the consequences for the host health. Abbreviations—AMPs: antimicrobial peptides; IgA: immunoglobulin A; NF-kB: nuclear factor kappa-light-chain-enhancer of activated B cells; STAT3: signal transducer and activator of transcription 3; Th17: T helper 17 cells; TLRs: toll-like receptors.

The gut microbiota plays a fundamental role not only for digestive equilibrium, but also for immunologic, hormonal, and metabolic homeostasis, as well as susceptibility to tumorigenesis [1,79]. While a state of homeostasis and symbiotic relationships is maintained by the separation of microbial entities from the host through mucosal–surface barriers, perturbation of this balance results in chain reactions, i.e., immune responses that ultimately emerge into a cancer-promoting status including qualitative and sometimes quantitative changes in the microbiota, rupture of the barrier at the level of tight junctions or of the mucous layer, and the consequent failure at the level of antibacterial defense systems, which overall evolve in the tumor microenvironment development [133,134]. Briefly, the mechanisms by which microbes could contribute to carcinogenesis include (reviewed in [133,134,135]):

1. DNA damage, both directly by inducing double-strand breaks and indirectly by eliciting high levels of ROS and RNS released from inflammatory cells, such as macrophages, as in colorectal cancer;
2. β-Catenin signaling alterations, as observed in gastric cancer and colorectal adenomas and adenocarcinomas, leading to upregulation of genes involved in cellular proliferation, survival, and migration, as well as angiogenesis;
3. Pro-inflammatory reactions induced by microorganism-associated molecular patterns that activate TLRs in several cell types, including macrophages, myofibroblasts, epithelial cells, and tumor cells, which, in loop, stimulate NF-κB, a master regulator of cancer-associated inflammation [136];
4. Activation of the IL-23–IL-17 axis, which promotes tumor growth and progression [137], tumor necrosis factor–alpha (TNF-α)–TNF receptor signaling, and IL-11–IL-6 family member signaling, all activating the signal transducer and activator of transcription 3 (STAT3) (belonging to the family of STAT transcriptional factors that participate in the regulation of a variety of cellular process, including proliferation, differentiation, inflammation, and stemness) [138,139,140];
5. Perturbations of the equilibrium between diet–gut microbiome–bile acid pool size/composition through the production of microbially generated secondary bile acids, like deoxycholic acid (DCA), responsible for activation of mitogenic and proinflammatory effects and subsequent promotion of liver cancer (see later in the text) [141,142] (Figure 2).

Of particular interest is the crosstalk between autophagy, a highly conserved catabolic process observed in eukaryotes, involving the formation of double-membrane vesicles called autophagosomes that engulf misfolded or aggregated proteins and damaged organelles for delivery to the lysosome, and microorganisms [143,144]. Autophagy, generally thought of as a survival mechanism [143], plays a relevant pathophysiological role in various disease processes, including cell death [145], infection [146], cardiovascular disease [147], neurodegenerative disorders [148], autoimmune diseases [149], and cancer [150]. As for cancer, autophagy (divided into five stages; see [150] for details), if, on the one hand, it promotes cancer by providing metabolites needed for the growth of tumor cells, on the other hand, it may also inhibit cancer progression by increasing apoptosis and, consequently, reducing the threats related to oxidative stress, persistent inflammation, and DNA damage [144]. Certain bacteria, like Hp, *Fusobacterium nucleatum*, and *Porphyromonas gingivalis*, have been demonstrated to regulate tumor formation and development by inducing or suppressing autophagy through a variety of mechanisms, including regulation of microRNA expression; production of inflammatory cytokines, like IL-6; and induction of G1 cell cycle arrest [144,151,152,153,154]. Therefore, the use of drugs that simultaneously target both autophagy and microbial communities (e.g., combined use of antibiotics, such as rapamycin, and autophagy inhibitors, such as chloroquine, [155]) can potentially represent a winning strategy to prevent or treat human cancer [144,150].

The microbiota also mediates tumor suppressive effects [133] through the inactivation of carcinogens, including the metabolism of certain hormones (e.g., reducing the risk for development of estrogen-dependent cancers [156]); the generation of SCFAs, which have been implicated in protection from colon and liver cancer [157,158,159]; and the biological activation of cancer-preventing phytochemicals, such as the major dietary polyphenols [160]. The specific aspect of the interaction between dietary substances, specifically, antioxidant nutrients, and the gut microbiota and the effects on various diseases, possibly including TC, will be discussed in Section 7.

### 5.1. Gut Microorganisms: A Defense against Cancer

The crosstalk between the microbiota and the immune system at the intestinal level is extensive and critical, with commensal organisms influencing the immune system both locally within the gut mucosa, by draining mesenteric lymph nodes, and systemically, preventing invasion and infection by opportunistic bacteria [57]. Mucus represents the primary barrier that limits contact between microbiota and host tissues and prevents microbial translocation [161]; thus, intestinal dysbiosis may lead to malfunction of the epithelial barrier and development, and local and general disorders may develop. Therefore, it should not be surprising that, in addition to its role in carcinogenesis, one emerging translational application of the gut microbiota is represented by its potential as a diagnostic and/or prognostic biomarker and, to consider its influence on cancer therapy efficacy, being implicated in mediating the therapeutic response and modulating cancer therapy toxicity (reviewed in [50,162]).

As reported in Section 5, once mucosal surface barriers are breached, microbes can further influence immune responses in evolving tumor microenvironments by stimulating proinflammatory or immunosuppressive pathways, although gut–tumor microenvironment crosstalk, especially in non-gastrointestinal cancers, has yet to be fully clarified [30,134]. Indeed, the gut microbiota contributes to immunoregulation via multiple and complex mechanisms within the gut-associated lymphoid tissue, which represents the largest component of the immune system (reviewed in [57]) (Figure 2):

- Goblet cells, specialized epithelial cells, are essential to the formation of the mucus barriers [163];
- Paneth cells, secretory cells located in the crypts of Lieberkühn, produce antimicrobial peptides (AMPs) and proteins [164], whose activity is enhanced following signaling from local immune cells in response to the microbiota;
- Upon recognition of microbial peptidoglycan, nucleotide-binding oligomerization domain-containing protein 2 (NOD2) contributes to intestinal homeostasis by molecular signaling through the kinase receptor-interacting protein 2 and NF-κB, and inducing the production of AMPs and mucin [165];
- Microbial metabolites activate pathogen recognition receptors (PRRs), such as NOD-, leucine-rich repeat- and NOD-like receptor family pyrin domain-containing 6 (NLRP6) inflammasome (having a role in the maintenance of a stable microbial community in the intestine), which lead to the secretion of IL-18 and AMPs [165];
- Microbial products also activate TLRs (i.e., TLR5), expressed in intestinal epithelial cells and belonging to the family of PRRs [166], which play a key role in the activation of innate immunity [167];
- Within adaptative mechanisms of microbial regulation, immunoglobulin A (IgA), secreted by plasma cells, terminally differentiated B cells residing in the intestinal lamina propria [168], acts primarily by inhibiting bacterial adherence to epithelial cells, also having direct effects on bacterial virulence [169];
- Pathogen-associated molecular patterns (e.g., LPS, flagellin) induce antigen-presenting cells, like dendritic cells (DCs); DCs then travel to mesentery, where they stimulate naïve T cells to differentiate into CD4+ Tregs and Th17 cells, which can migrate back to the gut mucosa or enter systemic circulation. While Tregs secrete the anti-inflammatory cytokine IL-10, Th17 cells, through the production of IL-17, can increase the Paneth cell production of antimicrobial peptides;
- SCFAs influence the immune response by a variety of signaling pathways, both in the innate immunity, e.g., activating NLRP3 that is responsible for IL-1β and IL-18 secretion [170]; inhibiting the production of cytokines, chemokines, and calprotectin produced by neutrophils [171]; inducing antimicrobial activity in the intestinal macrophage and increasing resistance to enteropathogens, and in the adaptative immunity, e.g., promoting the proliferation of Tregs [172]; and accelerating cellular metabolism and regulating gene expression to promote B cell differentiation into antibody-producing cells [173].

As described above, the combination of the epithelial barrier, mucus layer, IgA, and DCs and T cells shapes the ‘mucosal firewall’ [161]. Conversely, a dysbiotic microbiota can impact the host immune system through various mechanisms embracing the modulation of inflammasome signaling through microbial metabolites, the modulation of TLR signaling, and the degradation of secretory IgA [165] (Figure 2).

## 6. Diet: Enemy or Friend of the Gut Microbiota?

According to the World Health Organization, a healthy diet (vegetables, fruits, cereals, milk products, fresh meat) helps protect against malnutrition in all its forms, as well as noncommunicable diseases, including diabetes, heart disease, stroke, and cancer [174]. In the last decades, globally balanced diets (e.g., Mediterranean or traditional Asian diets) enriched with plant-based foods and poor in animal-based foods, particularly fatty and processed meats, have been progressively replaced by Westernized diets, characterized by high levels of fatty and processed meats, saturated fats, refined grains, salt, and sugars, but lacking in fresh fruits and vegetables [175,176]. Nutritional habits associated with a Western-style diet represent modifiable factors related to an increased risk of developing hypertension, hypercholesterolemia, overweight/obesity, and inflammation, which in turn imply a higher risk for disease conditions, such as metabolic syndrome, diabetes, cardiovascular disease, and cancer [175,176]. Notably, current evidence suggests a relevant role for obesity as a risk factor for differentiated TC [177,178], with no apparent effect on cancer aggressiveness [179,180]. Furthermore, the few studies conducted with the aim of exploring the association between dietary habits and the risk for TC highlighted that while dietary patterns of fruits, vegetables, seafood, and milk and dairy products led to a reduced risk of TC [10,181,182], the Western dietary pattern—starchy foods, sweets, and products rich in salt and fat—showed a significantly positive association with TC risk [10,183]. In contrast, the EPIC study, conducted on over 450,000 men and women from nine European countries for a total of 712 cases of differentiated TC identified over a 14.1-year follow-up, did not find a significant association between high adherence to a Mediterranean diet and decreased risk of differentiated TC [184].

Dietary components have been established to exert a significant impact on the structure and function of host gut microbial communities [19], and, as discussed in Section 5, alteration of the gut composition triggers a wide range of complex mechanisms that can pave the way for inflammation and, ultimately, tumorigenesis. In fact, despite substantial inter-individual variations in the composition of the microbial community, diet lifestyles have an important effect on the gut microbiota, being able to rapidly alter the microbial community structure and overcoming inter-individual differences in microbial gene expression [185]. In particular, while the increased count of the *Bacteroides* enterotype and the concomitant decrease in the abundance of *Bifidobacterium* are highly associated with animal protein and saturated fats, which characterize the Western diet, the enhanced abundance of Lachnospiraceae, Prevotallaceae, and *Bifidobacterium* and the reduction of *Lactobacilli* are induced by the consumption of high values for carbohydrates and simple sugars and the long-term intake of fibers, more typical of agrarian societies [186,187,188]. The composition of the gut microbiota is directly related to ROS production in the host; thus, an imbalance between the generation of ROS and antioxidant systems promotes the disruption of host gut microbiota homeostasis [19]. If, on a short-term scale, the consumption of food leads to certain levels of oxidative stress and inflammation through the activation of NF-κB that mediates the release of inflammatory cytokines (TNF-α, IL-6) and acute phase reactants (C-reactive protein—CRP) [189], Westernized diets, characterized by high caloric intake (high fat and/or sugars), might trigger an increase in the activity of mitochondrial respiration, with consequent overproduction of mitochondrial ROS and generation of chronic levels of inflammation [176,190].

Also, numerous chemical substances absorbed with food, generated in the gut by endogenous enzymatic activities, or produced by the action of bacterial metabolism have been linked to the development of cancers, especially of the intestine [29]. A high-protein diet is associated with the intake of N-nitroso compounds (NOCs), a class of chemical compounds that may react with DNA to form covalent addition products (DNA adducts), playing a central role in carcinogenicity if not repaired [141,191]. N-nitrosodimethylamine, one of the NOCs found in human food, predominantly in processed/cured meats and smoked/salted fish, is positively associated with colorectal cancer [192,193], while dietary nitrite, contained in processed and smoked food, is linked to gastric and esophageal cancers [193]. Of note, NOCs can also be formed endogenously in the gastrointestinal tract through the reaction of nitrite, previously produced by the reduction of nitrate (ingested with vegetables), with amines, amides, and other nitrosation precursors [194]. On the other hand, high-fat diets cause an increase in bile secretion and a higher fecal concentration of secondary bile acids (DCA and lithocholic acid are the most common), which are produced from gut microbial fermentation of primary bile acids via the 7α-dehydroxylation reaction [195,196]. DCA may in turn determine phylum-level alterations in the composition of the gut microbiota, accompanied by an impaired intestinal barrier and low-grade bowel inflammation, favoring the growth of opportunistic pathogens, such as *Ruminococcus*, *Shigella*, *Desulforvibrio*, and *Dorea*, which are recognized as key in the development of gut tumors [197]. Furthermore, DCA itself may induce genomic instability via several mechanisms, for example, giving rise to oxidative damage to DNA and damage to mitochondria and the endoplasmic reticulum, promoting the occurrence of colorectal cancer, inhibiting the apoptosis of cancer cells, and enhancing the progression and ability of metastasis of cancer cells [24,197]. Notably, altering the gut microbial composition through the growth of pathogenic bacteria results in pro-inflammatory effects (e.g., increased expression of pro-inflammatory IL-23 and suppressed expression of anti-inflammatory IL-10), and the associated loss of barrier function may lead to bacterial translocation, further driving pro-inflammatory effects, thereby increasing colorectal carcinogenesis [141].

In Section 5.1, we have highlighted the beneficial effects of SCFAs on the immune system. These metabolites are generated by microbial fermentation of non-digestible carbohydrates (non-starch polysaccharides, resistant starch, and soluble oligosaccharides like fructo-oligosaccharides); therefore, SCFAs are mainly produced through saccharolytic fermentation of carbohydrates that escape digestion and absorption in the small intestine [198,199]. Acetate, propionate, and butyrate account for more than 95% of SCFAs, at an estimated ratio of approximately 3:1:1 in the gut, although these relative proportions may vary with diet, microbiota, host genotype, and the site of fermentation [200]. While acetate production pathways are widely distributed among enteric bacteria and acetogens, the main propionate production pathway is used by Bacteroidetes and certain Firmicutes genera [141,199]. Conversely, only a small number of organisms, e.g., *Faecalibacterium prausnitzii*, *Eubacterium rectale*, *Eubacterium hallii*, and *Roseburia* spp., appear to be responsible for the major fraction of butyrate production [141,201]. SCFAs modulate the immune response by a variety of signaling pathways. The binding of SCFA to the G protein-coupled receptors (GPRs: GPR41, GPR43, and GPR109A) on the surface of colonocytes and immune cells inhibits the release of the pro-inflammatory cytokines IL-1β and IL-6 by regulating the upstream NF-kB pathway [66,141,202]. Additionally, SCFAs are natural inhibitors of HDACs that, deacetylating lysine in the histone, promote gene repression/silencing [66,203]. Through histone deacetylation, SCFAs may inhibit NF-kB activity, resulting in anti-inflammatory effects, namely, increased production of IL-10 and downregulation of pro-inflammatory molecules IL-12, TNF-a, IL-1β, and NO [66,204,205]. In contrast, inhibition of HDACs results in an increase of MAPK signaling and proinflammatory cytokine secretion. Similarly, SCFAs can participate in pro-inflammatory effects by activating GPR41 and GPR43 receptors, which, inducing extracellular signal-regulated kinases (ERK) 1, 2 and p38 MAPK phosphorylation, respectively, further increase the production of pro-inflammatory factors [66,206,207]. Therefore, current evidence indicates that dietary fibers increase the production of SCFAs, which, although representing only a small part of the wide range of metabolites produced by gut bacteria, have relevant effects in protecting the colon barrier function and in influencing health maintenance and disease development (e.g., inflammatory bowel disease, obesity, diabetes, kidney and liver diseases, hypertension, cancer) [200,208].

## 7. Antioxidant Nutrients at the Intersection of Gut Microbiota Health and Protection against Thyroid Cancer

In the previous sections, we discussed how an imbalance of ROS production, in addition to being a signature of TC, does affect the health of the gut microbiota. Indeed, oxidative stress is responsible for a direct effect on the thyroid gland, as well as substantial changes in the microbial composition (increased abundance of Firmicutes and *Bacteroides* and decrease in *Lactobacillus*, *Roseburia*, and *Blautia*) and a reduction in microbial diversity [19], which in turn could be linked to TC development. Thereinafter, we will describe the effects of major categories of nutrients that may influence both TC risk and gut microbiota health.

### 7.1. Probiotics

Probiotic bacteria are living nonpathogenic microorganisms (*Lactobacillus* and *Bifidobacterium* the most used probiotic genera, overall named as LAB), which, when administered in adequate amounts, confer microbial balance. Particularly in the gastrointestinal tract, they have been demonstrated to exert antioxidant capacity in different ways [209]. Probiotics from the LAB family can be potential candidates to produce functional foods or natural antioxidant supplements due to their own antioxidant enzymatic systems (superoxide dismutases—SODs, which catalyze the breakdown of superoxide into H_2_O_2_ and water, and CAT, which decomposes H_2_O_2_) and the ability to chelate ferrous and cupric ions [209,210]. Moreover, probiotics can produce various metabolites with antioxidant activity, such as glutathione (GSH), butyrate, and folate, the latter involved not only in the process of DNA-biosynthesis, with implications for genomic repair and stability, but also in the antioxidant activity, e.g., scavenging free radicals, such as peroxynitrite, and inhibiting lipid peroxidation [209,211]. Probiotics can also stimulate antioxidant host defenses, increasing SOD, CAT, and GPx concentration, and downregulating expression of enzymes, such as NOX, cyclooxygenase, and cytochrome P450 superfamily, implicated in ROS generation [209]. Probiotic supplementation also influences thyroid function. SCFAs, especially butyric acid, inhibit HDAC and activate re-expression, thereby enhancing iodine uptake [32]. Furthermore, *Lactobacillus* promotes the uptake of Se that, increasing PAX8 expression and its DNA-binding capacity, might enhance NIS expression and activity, the latter being silenced in most BRAF^V600E^-positive PTC [212]. Interestingly, probiotic supplementation composed of *Bifidobacterium infantis*, *Lactobacillus acidophilus*, *Enterococcus faecalis*, and *Bacillus cereus* alleviated complications and restored the gut and oral microbial diversity in TC patients [213].

### 7.2. Trace Elements

Se, an essential micronutrient naturally contained in seafood, cereals, and dairy products and available as a dietary supplement, is incorporated as selenocysteine in 25 selenoproteins in humans, most of which are functionally characterized, including oxidoreductases, which have been proved to be effective in redox regulation and antioxidant activity [214,215]. Additionally, Se exerts immunomodulatory effects, participating in differentiation of naive CD4+ Th cells into two subgroups [216]. While Se deficiency has been linked to Th2 cell response (involved in antibody-mediated immunity), higher Se levels favor Th1 cell response (implicated in cell-mediated immunity) [15,216]. The thyroid gland is particularly rich in Se, which takes part in the structure of antioxidant enzymes (e.g., GPx, TrxR), as well as the three deiodinases, and Se deficiency has been associated with the development of autoimmune thyroid diseases [69]. Se levels were significantly decreased in patients with PTC and FTC [217], although, so far, there is no evidence of an association between overall Se intake (diet plus supplementation) or dietary Se intake and the risk for TC [218,219]. Furthermore, Se dietary intake and supplementation improve the diversity of the microbiota, causing a decline in *Parabacteroides* belonging to the phylum Bacteroidetes [220], and increasing the relative abundance of some health-relevant taxa (e.g., the families Christensenellaceae and Ruminococcaceae and the *Lactobacillus* genus) [221].

Zn, mainly present in lean meat, eggs, seafood, beans, nuts, and chickpeas, is known as an antioxidative trace element due to its role as a cofactor in SOD [15,26]. Zn also plays a key role in the metabolism of thyroid hormones, specifically by inhibiting hepatic D1 and acting as a cofactor of D2, regulating the synthesis of TSH and of thyrotropin-releasing hormone, as well as by modulating the structures of essential transcription factors involved in the synthesis of thyroid hormones and in thyroid hormone receptors [222]. Importantly, Zn could participate in the carcinogenic process of TC, as suggested by an increased copper/Zn ratio in serum and thyroid tissue of patients with TC in comparison to healthy controls or patients affected by benign thyroid diseases [223,224]. As regards the effects on the gut microbiota, they may vary, with beneficial or negative consequences for microbial abundance and key processes for survival, depending on the source of exposure (e.g., zinc oxide, zinc sulfate) and the species [225].

### 7.3. Vitamins

Growing evidence indicates that vitamin D, a secosteroid hormone, has beneficial effects on several body systems other than the musculoskeletal system [226]. It is mostly synthesized in the skin through ultraviolet irradiation (vitamin D3 or cholecalciferol, also found in animal-sourced foods, such as fatty fish—trout, salmon, tuna, and mackerel—fish liver oil, and egg yolk), and only 5–10% is taken from food (both vitamin D3 and vitamin D2 or ergocalciferol, contained in mushrooms) [227,228]. Vitamin D could be considered an adjuvant therapy for relieving inflammation and oxidative stress [229]. Indeed, it has potent anti-inflammatory properties, contributing to a reduction in pro-inflammatory mediators (IL-6, IL-8, IL-9, IL-12, TNF-α, CRP, and interferon gamma) and an increase in anti-inflammatory cytokines, such as IL-10, IL-5, and IL-4 [228,229,230,231]. Furthermore, vitamin D supplementation might increase serum levels of total antioxidant capacity and GSH and significantly decrease the concentration of malondialdehyde, derived from the peroxidation of polyunsaturated fatty acids and commonly used as a marker of oxidative stress [232,233]. While vitamin D deficiency appears to be associated with microbiota dysbiosis, interventional studies reported that vitamin D changes the microbiota composition, leading to increased beneficial bacteria, such as Ruminococcaceae, *Akkermansia*, *Faecalibacterium*, *Lactococcus*, and *Coprococcus*, and decreasing some genera from Firmicutes [234]. Although the effects of vitamin D on thyroid function still remains to be fully established [35,228], a meta-analysis based on 14 studies showed that vitamin D deficiency could act as a risk factor for TC [235]. Also, a recent study reported that vitamin D supplementation was positively associated with all-cause and total cancer mortality in differentiated TC and might be a modifiable prognostic factor for improved survival [236].

Vitamin C, also known as L-ascorbic acid, an essential nutrient that cannot be synthesized by humans due to loss of a key enzyme in the biosynthetic pathway, is naturally present in a wide variety of fruit and vegetables, added to others, and available as a dietary supplement [26,237]. Vitamin C is a potent hydrophilic antioxidant capable of scavenging numerous reactive species (H_2_O_2_, singlet oxygen, peroxide radicals, and hydroxyl radicals) and of regenerating cellular and membrane antioxidants, like GSH and vitamin E [237,238]. Moreover, vitamin C has been shown to enhance neutrophil migration in response to chemoattractants and phagocytosis of microbes, and support caspase-dependent apoptosis, inducing uptake and clearance by macrophages, which, overall, result in supporting the resolution of the inflammatory response and attenuating tissue damage [237]. On the other hand, vitamin C may undergo the Fenton reaction, reducing metal ions, like trivalent iron and copper ions, thus promoting a reaction that gives rise to highly reactive free radicals [239]. This pro-oxidant activity seems to be more relevant in tumor cells, where the anti-tumor activity of vitamin C may lead to DNA, protein, and lipid damage [240]. In this framework, through ROS-dependent mechanisms, vitamin C was found to trigger cytotoxicity in PTC cells [241], suppress the MAPK/ERK and phosphatidylinositol 3′-kinase/AKT signaling pathways in BRAF wild-type or mutant thyroid cancer cells [242,243], and induce ferroptosis in ATC cells [244], suggesting a potential strategy for TC therapy. In addition, vitamin C therapy has beneficial effects on the gut microbiota by significantly increasing the α-diversity of microbial communities in the intestine, as well as the ratio of Firmicutes/Bacteroidetes both in humans and animal models [245].

### 7.4. Polyphenols

Polyphenolic compounds, plant secondary metabolites further classified into four main classes (flavonoids, phenolic acids, lignans, and stilbenes) based on their chemical structure, are widely distributed in plant-based foods, such as tea, coffee, wine, fruit, vegetables, whole-grain cereals, and cocoa [246]. Polyphenols are the most abundant antioxidants in the diet, acting as chelators, donating electrons or hydrogen to reactive oxygen, nitrogen, and chlorine species, therefore inhibiting the formation of unstable radicals and scavenging and intercepting free radicals [16,247]. The gut microbiota plays a fundamental role in the metabolism of dietary polyphenols that in turn affect the microbial composition [248]. In particular, polyphenolic compounds protect the function of the gut, influence mucus and antimicrobial peptide secretion; exert anti-inflammatory effects modulating the production of cytokines and immunoglobulins; and regulate molecular signaling, such as the NF-κB pathway, which triggers the inflammatory response [248]. Moreover, supplementation of tea polyphenols may restore the richness and diversity of the gut microbial population, also regulating gut dysbiosis and increasing the relative abundance of beneficial microbes, like *Lactobacillus*, *Akkermansia*, *Blautia*, *Roseburia*, and *Eubacterium* [249]. At the same time, a polyphenol diet intervention can selectively inhibit the growth of pathogenic bacteria [250]. While grape pomace promotes the decrease of Enterobacteriacae and *Escherichia coli* [251], a combination of quercetin and resveratrol leads to a reduced relative abundance of Desulfovibrionaceae, Acidaminococcaceae, Coriobacteriaceae, *Bilophila*, and Lachnospiraceae (all possibly linked to diet-induced obesity) [252]. As regards the relationship with TC, although polyphenols might target certain molecules, such as NADPH oxidases (including DUOX1,2 and NOX4), TPO, and Nrf2 (an important mediator of the antioxidant defense) related to oxidative pathways in thyroid glands, there is no current evidence of a possible association between the intake of any polyphenol class and the risk of differentiated TC [16,253]. However, an inverse association was observed between polyphenols and phenolic acid intake and differentiated TC risk in subjects with a body mass index ≥ 25, indicating protective associations in overweight and obese TC patients [253]. In addition, a nested case–control study in women showed that blood polyphenol concentrations were mostly not associated with TC risk, except for high blood concentrations of caffeic, 3,4-dihydroxyphenylpropionic, and ferulic acids, which were reported to be related to a lower risk of PTC [254].

## 8. Promoting Food Health: Should Citizens and Food Companies Meet Half Way?

It is now widely accepted that the attitude towards certain types of food, particularly among youngest people, the most important recipients of policies oriented to healthy food benefits, depend not only on the nutritional values of these compounds, but also, and primarily, on the packaging and branding such compounds are included in. Already some time ago, when social media and the Internet were not popular, it was demonstrated that even a single exposure to a television-based advertisement, less pervasive than actually occurring with the modern communication era, was capable of affecting the brand preferences of children [255]. Later on, but still back some 15 years ago, the same group found that the branding of foods and beverages influences the taste perception of young children, with the same products that, when marked with the brand of a popular fast-food company, were selected with respect to control food by a cohort of children [256]. However, this kind of reaction is mainly driven by physical senses, mainly vision and somewhat touch, interrelated with memory cues; although very important also in maintaining the human being in life, safety, and security, physical senses are just a portion of our sensory system, the other one being represented by chemical sensoriality, i.e., smell and taste.

Smell and taste are the two sensory modalities with which humans enter in closer relationships with edible compounds; thus, they are capable of eliciting emotional attributes for objects or events, influencing our mood, thoughts, or even fostering or repressing our social interactions [257,258]. As such, although being not straightforward or timely, like other sensory modalities, the representation of an edible compound with peculiar olfactory and gustatory characteristics might have an important role in the education of individuals towards safe and positive dietary habits, in turn promoting the healthy lifestyle of a community overall. The process is not easy, also because olfactory or gustatory preferences are quite subjective, and a complete customization of a given product could be required to attain a full penetration into a reference market; however, strong common traits in this regard are present in culturally uniform populations or groups, and, to a wider extent, it is thought that major universal drivers are present throughout the globe, modulating a quite uniform reaction to certain chemosensory stimuli [259].

**Figure 3:**
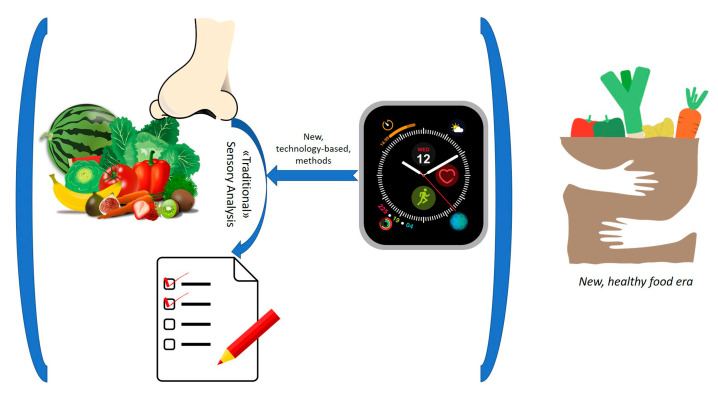
A technology-based paradigm of sensory analysis to build up new foods for a novel healthy food era.

In this framework, when conceiving and distributing a product on the shelf, major companies usually perform well-structured, exhaustive sensory assessments, relying on the wide experience and expertise with this kind of task of experienced referees (or panelists). The approach commonly adopted is based on validated questionnaires groups of judges are asked to complete within a structured smelling/tasting session within a well-defined, structured environment. Although well accepted by the scientific community and by expert referees, this methodology hides some methodological limitations, including judgment biases, also among experts; therefore, an attempt should be made to establish a positive synergy between traditional and innovative approaches to (chemo-)sensory analysis. This should be made considering the role of psychophysiological responses of panelists to sensory stimuli [260], even when measured with consumer technologies, including wearable sensors for the characterization of brain and autonomic responses of the human beings. However, the responses provided by trained referees can only represent a first step towards the implementation of edible, healthy compounds characterized by valuable sensory properties and is probably scarcely representative of the success a given product can achieve on the market. As such, specific features of psychophysical responses can be sought in consumer groups based on the physiological data eventually collected by personal technological gadgets in semi- and non-structured scenarios, with the pivotal support of Artificial Intelligence models for data analysis and interpretation. Therefore, the so-called Internet-of-Everything paradigm can be leveraged in this scenario, and the benefits of its application, already largely demonstrated in the neuromarketing (for a review, see [261]), can effectively drive towards a new era of nutrition, where novel foods, merging important characteristics in terms of being healthy, presenting excellent nutritional values, being environmentally sustainable, as well as palatable and appealing from the sensory point of view, can be released in the market and produce short- and long-term benefits for the citizenship in terms of healthy lifestyle promotion and reducing the likelihood of the onset of important health conditions (e.g., cardiovascular, neurological, and oncological diseases), acting as a modifiable factor in this regard [182] (Figure 3).

## 9. Conclusions

While a growing body of evidence supports the influence of the gut microbiota on the thyroid function, with microbial communities and their metabolites interacting with molecules and enzymes involved in thyroid hormone synthesis and, through immunomodulatory effects, possibly increasing the risk of autoimmune thyroid disease, recent studies indicate that microbiota dysbiosis may participate in the thyroid carcinogenesis process. Not only intestinal microorganisms, through immunomodulatory actions, may trigger subsequent inflammation and oxidative stress in other body districts, including the thyroid gland, but emerging research has reported the presence of microbial communities within the thyroid tumor microenvironment, which could promote TC progression and severity, as observed in other extra-gut tumors. In particular, the abundance of certain bacteria genera may represent a prognostic value, also making it possible to distinguish tumor from peritumor tissues and across different TC subtypes. Therefore, the tumor microbiome potentially represents a novel strategy against TC since targeting specific microorganisms by appropriate techniques might improve the effectiveness of traditional treatments and contribute to the advancement of oncology care. Obviously, these findings, since obtained from a limited number of countries, need to be verified within multi-center longitudinal studies with a larger sample size, which further explore close and direct correlations between microbial alterations, including the oral microbiota, and TC development across a more diverse patient population, with the aim of identifying the most appropriate therapy possible, personalized for each patient. Also, while most bacterial communities found in the tumoral milieu are microbiomes commonly present in the gut, the translocation of bacteria from the gut to TC still requires further exploration in animal models or functional research in vitro.

The intake of antioxidant nutrients may help maintain the gut microbiota in dynamic equilibrium with the host, since specific classes of substances, like essential micronutrients, vitamins, polyphenols, and probiotics, themselves promote anti-inflammatory effects and counteract redox status imbalance. Although no definite association between dietary factors and TC has been currently fully established, antioxidant nutrients might reduce the risk for TC, whose pathogenesis appears to be mainly regulated by oxidative stress and downstream-related pathways. Thus, while some eating habits and overweight/obesity increase the risk of developing TC, reduced consumption of fat/sweets and/or antioxidant nutrient supplementation could be useful as adjuvant and/or customized therapy in controlling thyroid homeostasis and the metabolic state of the whole body. Relying on such assumptions, leveraging new technologies and tools actually available on the market, the scientific community should engage in developing new foods with high nutritional value, antioxidant capability, and intriguing features in the sensory domain to promote a healthy lifestyle based on nutrition and diminishing the likelihood of developing cancer or other burdensome diseases, at least through the modulation of the so-called modifiable factors. Future studies investigating the association between environmental exposure, dietary patterns, metabolism, gut microbial composition, and TC carcinogenesis would improve the understanding of TC etiopathogenesis, as well as the bidirectional relationship between diet and the microbiota in the human gut.

## Figures and Tables

**Table 1:** Main characteristics of the human studies investigating the association between the gut microbiome and thyroid cancer.

Study Design	Sample	Country	Microbial Indicators	Other Clinical Data	Reference
Cross-sectional	Blood sample from 77 patients with PTC (37 patients in distant metastasis group and 40 in ablation group) after total thyroidectomy and radioiodine treatment	China	-	31 differentially abundant metabolites between the ablation and distant metastasis groups: 12 metabolites with higher concentrations in the distant metastasis group, while 19 metabolites showing lower concentrations. 31 metabolites mostly involved in “alanine, aspartate and glutamate metabolism” and “inositol phosphate metabolism”.	[71]
Case-control	Peripheral blood and fecal samples from 30 preoperative patients diagnosed for TC and 35 healthy controls, 18 to 65 years of age	China	Chao index (richness) and Shannon index (richness and evenness) higher in the TC group. Β-diversity (Bray–Curtis method) significantly different between the two groups. Firmicutes and Proteobacteria increase and Bacteroidetes decrease in TC group.	TC-enriched genera, like *Lactococcus*, *Ruminococcaceae_UCG_002*, *Intestinibacter*, *Clostridium_sensu_stricto_1*, *Christensenellaceae_* *R-7_group*, *Lachnospiraceae_ND3007*, and *Turicibacter*, positively correlated with Lpa titers. TC-enriched genera, including *Ruminococcaceae_UCG-014* and *Lactococcus*, negatively correlated with the levels of ApoB. 72 significantly changed metabolites (mainly lipids) in TC group. Some TC-enriched genera, e.g., *Klebsiella*, *Coprococcus*_*3*, and [*Eubacterium]_hallii_group*, associated with lipid-related metabolites. The adipocytokine signaling, sphingolipid metabolism, cholesterol metabolism, and necroptosis pathways also significantly enriched in TC patients.	[20]
Case-control	Fecal specimens from 74 subjects: 20 patients suffering from differentiated TC, 18 patients with thyroid nodules, and 36 healthy controls	China	35 unique species, like *Thiobacillus*, *Rhodobacter*, *Rheinheimera*, *Mycobacterium*, and *Anaerotruncus*, found in TC group. Shannon index not significant. ACE index (microbial abundance) higher in TC and thyroid nodule groups than in control group. UniFrac distance (β-diversity) significantly different between thyroid disease and healthy groups. The gut microbiome of TC group characterized by a dominance of *Prevotella*, *Roseburia*, *Coprococcus*, *Anaerostipes*, *Ruminococcus*, *Neisseria*, *Streptococcus*, and *Porphyromonas.*	In TC group, relationships between 22 taxa and 7 clinical indices: significantly positive associations observed between TSH and the genus *Porphyromonas* and between FT3 and the genus *Streptococcus*, and significantly inverse associations between TRAb and the families Clostridiaceae and Lachnospiraceae and between TPOAb and the genus *Ruminococcus.*	[59]
Cross-sectional	Stool samples collected prior to surgery from 90 patients with TC (88 cases with PTC and 2 cases with FTC; 56 cases of TCs with local lymph node metastasis—N1 group and 34 of TCs without local lymph node metastasis—N0 group) and 90 healthy controls	China	Reduced richness (Ace index) and diversity (Shannon index) in TC samples. Significant differences in β-diversity (UniFrac distance) between TC and healthy control groups. No significant difference in the Firmicutes/Bacteroidetes ratio between the two groups. Relatively higher abundance of Proteobacteria in TC patients.	No significant difference in Ace and Shannon indices between N0 and N1 subgroups. A four-genus microbial signature able to distinguish TC patients in N0 from those in N1 (*Hungatella*, *Alistipes*, *Fusobacterium*, and *Phascolarctobacterium*). Five categories related to the processing of genetic information (‘‘Aminoacyl-tRNA biosynthesis,” ‘‘Homologous recombination,” ‘‘Mismatch repair,” ‘‘DNA replication,” and ‘‘Nucleotide excision repair”) significantly increased in the healthy controls compared to TC patients.	[79]

Abbreviations—ApoB: apolipoprotein B; FT3: free triiodothyronine; FTC: follicular thyroid cancer; Lpa: lipoprotein a; PTC: papillary thyroid cancer; TC: thyroid cancer; TPOAb: thyroid peroxidase antibody; TRAb: thyroid receptor antibody; TSH: thyroid-stimulating hormone; UPLC-Q-TOF/MS: ultraperformance liquid chromatography quadrupole time-of-flight tandem mass spectrometry.

**Table 2:** Main characteristics of the human studies investigating the association between the tumor microbiome and thyroid cancer.

Study Design	Sample	Country	Microbial Indicators	Other Clinical Data	Reference
Cross-sectional	25 patients with TC (19 malignant and 6 benign tumors), aged 25–66 years. Stool samples collected before thyroidectomy. Tissue samples removed during surgery and divided into tumor, paratumor, and normal tissues.	China	α-diversity (Chao1, Shannon, and Simpson indices) different in all types of samples. All fecal diversities higher than those in tissues. Diversity in malignant patients lower than that in benign patients, and decreasing with distance from cancerous tissues. Proteobacteria the most abundant in all three types of tissues, while Firmicutes dominant in stool samples.	The microbiome from thyroid completely different from that of the gut, with 18 of the 30 pathways significantly different. Two of the most abundant operational taxonomic units, *Pseudomonas mucidolens* and *Escherichia fergusonii*, associated with several processes. Several microbes negatively related with PTH, while *Neisseria perflava* positively related with TSH and T3.	[1]
Retrospective	Tumor and peritumor tissues collected from 30 patients with PTC who underwent total thyroidectomy.	China	Chao1 index (richness) and Shannon index (richness and evenness) lower in tumor tissues than in peritumor tissues (the first index not significant). β-diversity (Bray–Curtis method) significantly different between the two groups. At the genus level, abundance of *Sphingomonas* and *Aeromonas* significantly increased in tumor tissues, whereas the abundance of *Comamonas*, *Acinetobacter*, *Peptostreptococcus*, and *Proteus* significantly increased in peritumor tissues.	Chao1 index significantly higher in N1 stage (presence of lymph node metastasis) than in N0 stage (absence of lymph node metastasis); Shannon index significantly lower in N1 stage than in N0 stage. Abundance of *Sphingomonas* significantly increased in N1 stage compared to N0 stage. No significant difference in Chao1 and Shannon indices between male and female patients. No difference in the diversity and composition between patients <55 and ≥55.	[12]
Retrospective	Raw whole-transcriptome RNA-sequencing, Level 3 normalized mRNA expression read counts, and DNA methylation 450 k sequencing data for untreated, nonirradiated tumor, and adjacent normal tissue were downloaded from the Genomic Data Commons legacy archive for 563 TC patients (354 CPTC, 101 FVPTC, 35 TCPTC, 135 male, 366 female tumor samples: 505 tumor and 58 normal tissue samples).	United States	45 microbes in CPTC, 34 in FVPTC, and 33 in TCPTC differentially abundant between tumor and normal tissue. 33 microbes in male samples and 49 microbes in female samples to be differentially abundant between tumor and normal tissue. *Micrococcus luteus*, *Frankia* sp., *Anabaena* sp. *K119*, and *uncultured* *Gammaproteobacteria bacterium* all similarly overabundant in normal tissue in CPTC, FVPTC, and TCPTC. In males, *Synechococcus* sp. *CC9311* overabundant in the normal samples, while in females, overabundant in tumor samples.	*Frankia* sp. and *uncultured* *Gammaproteobacteria bacterium* *Gammaproteobacteria bacterium*, which are abundant in all PTC normal tissue samples, correlated with lower MACIS score). *Bradyrhizobium* sp. *BTAi1*, which is uniquely abundant in TCPTC normal tissue, correlated with higher MACIS score. *Frankia* sp. and *Anabaena* sp. *K119*, both of which are overabundant in normal tissue samples of all PTC subtypes, negatively correlated with pathologic M stage, while *Stenotrophomonas maltophilia, * dysregulated in only CPTC and FVPTC, positively correlated with pathologic M stage. Microbe abundance in FVPTC correlated with the greatest number of chromosomal alterations and mutations. Microbe abundance in males more frequently and strongly correlated with a greater number of CNV. Microbes dysregulated in TCPTC and male patients correlated with higher expression of the BRAF^V600E^ mutation. The majority of most enriched pathways uniquely dysregulated in the different subtypes related to cell growth, in males belonging to the tumor suppression-related group, and in females, belonging to the DNA checkpoint and damage-related group. Lower microbe abundance correlated with greater extent of methylation at known tumor suppressor genes.	[97]
Cross-sectional	Tumor samples from 80 patients with PTC.	China	α-diversity significantly lower in patients with T1/T2 PTC than in those with T3/T4 PTC (Shannon and Simpson indices). No significant differences in richness (Sobs index) between clinical stages. β-diversity (Bray–Curtis metric distances) significantly different with tumor progression, with differences in *Psudomonas*, *Rhodococcus*, and *Sphingomas* in abundance among various stages. *Pseudomonas* spp., presenting a higher abundance in tumors of patients with T1 and T2 PTC than in T3 or T4; *Rhodococcus* abundance significantly higher in T1 than in T3 PTC; and *Sphingomonas* more abundant in T1 and T2 than in T3. Eight-genera microbiome signature between T1_2 and T3_4 groups. α-diversity significantly lower in males compared to females, but no significant differences in β-diversity by sex. *Rhodococcus*, *Ralstonia,* *Chryseobacterium*, and *Burkholderia-Caballeronia-Paraburkholderia* more abundant in females than in males.	In T1_2 group, higher proportions of most metabolic pathways, while in T3_4 group, higher proportions of super pathways of purine nucleotide de novo biosynthesis and palmitate biosynthesis II. Positive associations between FT4 and *Neisseria* and *norank_f__norank_o__Chloroplast*, and FT3 and *Treponema*. Negative associations between FT4 and *Klebsiella*; T4 and *Klebsiella* and *Escherichia-Shigella*, T3 and *Granulicatella*; and TSH and *norank_f__norank_o__Clostridia_UCG-014* and *Prevotella.* A negative correlation between anti-T SHR levels and *Klebsiella* and *Burkholderia-Caballeronia-Paraburkholderia*. Positive correlation of the anti-TG levels with *Sphingomonas*, *Rhodococcus*, *Ralstonia,* and *Brevundimonas*, but negative correlation with *Anaerococcus* and *Akkermansia*. Nine genera (*UCG-002*, *Streptococcus*, *Parvimonas*, *Akkermansia*, *Bacteroides*, *Haemophilus*, *Selenomonas*, *Prevotella*, and *Bifidobacterium*) negatively correlated with the anti-TPO levels.	[82]

Abbreviations—CNV: copy number variation; CPTC: classical papillary thyroid cancer; FT3: free triiodothyronine; FT4: free thyroxine; FVPTC: follicular variant papillary thyroid cancer; MACIS: distant metastasis, patient age, completeness of resection, local invasion, and tumor size; PTC: papillary thyroid cancer; PTH: parathyroid hormone; T1–T4: used to identify the size and extension of the tumor, with progressive enlargement and invasiveness from T1 to T4; T3: triiodothyronine; TCPTC: tall cell papillary thyroid cancer; TC: thyroid cancer; TG: thyroglobulin; TPO: thyroid peroxidase; TSH: thyroid-stimulating hormone.

## Data Availability

Not applicable.

## Abbreviations

AITD	Autoimmune thyroid disease
AMPs	Antimicrobial peptides
ATC	Anaplastic thyroid cancer
CAT	Catalase
CRP	C-reactive protein
D1,,/3	Deiodinase type 1,2,3
DCs	Dendritic cells
DCA	Deoxycholic acid
DUOX	Dual oxidase
ERK	Extracellular signal-regulated kinase
FTC	Follicular thyroid cancer
GABA	Gamma-aminobutyric acid
FVPTC	Follicular variant of papillary thyroid cancer
GPx	Glutathione peroxidase
GPR	G protein-coupled receptors
GSH	Glutathione
H_2_O_2_	Peroxide hydrogen
HD	Hashimoto’s disease
HDAC	Histone deacetylase
HIF-1α	Hypoxia-inducible factor-1 alpha
Hp	Helicobacter pylori
IL	Interleukin
LPS	Lipopolysaccharide
MAPK	Mitogen-activated protein kinase
NF-kB	Kappa-light-chain-enhancer of activated B cells
NIS	Sodium iodide symporter
NLRP3,6	NOD-like receptor family pyrin domain-containing 3,6
NO	Nitric oxide
NOCs	N-nitroso compounds
NOD	Nucleotide-binding oligomerization domain-containing protein
NOX	Nicotinamide adenine dinucleotide phosphate oxidase
PAX8	Paired box 8
PDTC	Poorly differentiated thyroid cancer
PRRs	Pathogen recognition receptors
PTC	Papillary thyroid cancer
RNS	Reactive nitrogen species
ROS	Reactive oxygen species
rRNA	Ribosomal RNA
SCFAs	Short-chain fatty acids
Se	Selenium
SOD	Superoxide dismutase
STAT3	Signal transducer and activator of transcription 3
T3	Triiodothyronine
T4	Thyroxine
TAMS	Tumor-associated macrophages
TC	Thyroid cancer
TCPTC	Tall cell papillary thyroid cancer
TGF-β1	Transforming growth factor-beta 1
Th	T helper cell
TrxR	Thioredoxin reductase
TLR	Toll-like receptor
TNF-α	Tumor necrosis factor-alpha
TPO	Thyroperoxidase
TR	Thyroid receptor
TSH	Thyroid-stimulating hormone
Treg	Regulatory T cell
Zn	Zinc
